# Leadless pacemaker implantation with hybrid image mapping technique in a congenital heart disease case

**DOI:** 10.1016/j.hrcr.2021.07.004

**Published:** 2021-07-18

**Authors:** Jose Luis Martinez-Sande, Laila Gonzalez-Melchor, Javier Garcia-Seara, Moises Rodriguez-Mañero, Xesus Alberte Fernandez-Lopez, Jose Ramon Gonzalez Juanatey

**Affiliations:** University Clinical Hospital of Santiago de Compostela, CIBER-CV, Santiago de Compostela, Spain

**Keywords:** Congenital heart disease, Electroanatomical mapping system, Leadless pacemaker, Sinus venosus atrial septal defect, Ultrasound intracardiac imaging catheter

## Introduction

Sinus venosus atrial septal defect (SVASD) is an interatrial communication caused by a deficiency of the common wall between the superior vena cava and the right-sided pulmonary veins. Implantation of a conventional pacemaker in this setting can be challenging. We report the feasibility of leadless pacemaker (LPM) implantation assisted by imaging integration and electroanatomical mapping.

## Case report

**Figure 1:**
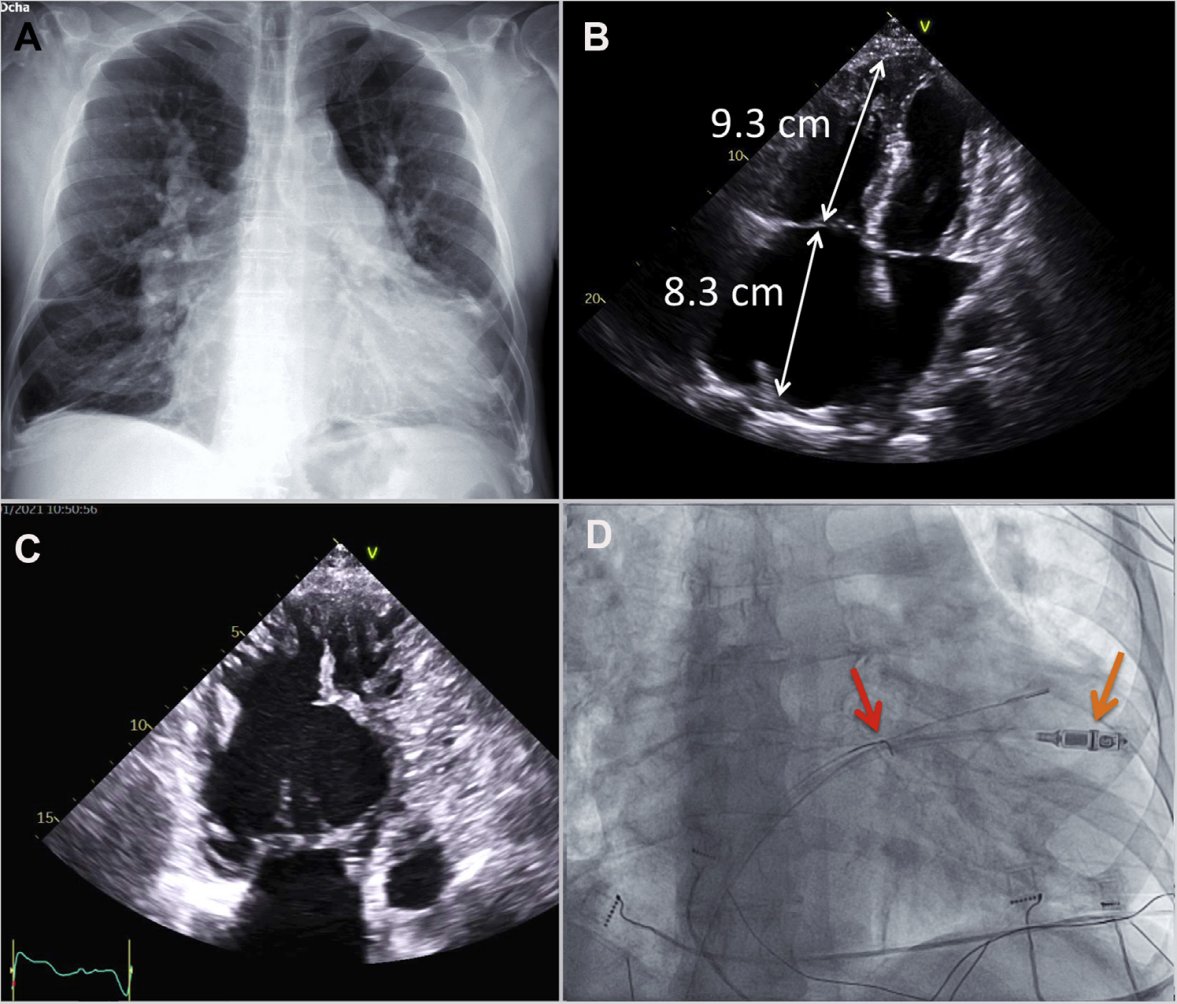
**A:** Posteroanterior thoracic radiograph with typical pulmonary arterial hypertension findings. **B:** Echocardiography 4-chamber view with severe right dilated chambers. Right ventricle: 9.3 cm. Right atrium: 8.3 cm. Right atrium area: 53.3 cm^2^. **C:** Modified apical 4-chamber view with right ventricle hypertrophic trabeculae. **D:** Fluoroscopic right anterior oblique view during leadless pacemaker (LPM) implantation with an Amplatz Gooseneck snare (Medtronic, Minneapolis, MN). *Red arrow:* Amplatz Gooseneck snare. *Orange arrow:* LPM deployment.

We present the case of a 79-year-old man with a recent diagnosis of unrepaired SVASD. The patient presented with a left atrial flutter with rapid ventricular response, which conditioned poor clinical tolerance and multiple episodes of heart failure decompensation. Three years ago, he underwent a cavotricuspid isthmus–dependent atrial flutter ablation, which was performed by means of an electroanatomical mapping system (CARTO; Biosense Webster, Diamond Bar, CA). Considering his multiple comorbidities and the severe biatrial dilation, he was now referred for LPM implantation plus atrioventricular (AV) node ablation. The patient had severe pulmonary arterial hypertension and the thoracic radiograph showed bilateral hilar prominence with marked cardiomegaly (Figure 1A). Transthoracic echocardiogram showed a left ventricular ejection fraction of 50% with dilated right chambers and multiple right ventricle (RV) hypertrophic trabeculae with severe tricuspid regurgitation. The anteroposterior RV diameter was 56 mm and the right atrium (RA) 80 mm; RA area was 48 cm^2^ (Figure 1B and 1C). Owing to this severe right chamber dilation (with the potential suboptimal deployment owing to insufficient catheter reach), ultrasound intracardiac imaging catheter (ICE) (SOUNDSTAR, Biosense Webster) combined with 3-dimensional (3-D) ultrasound reconstruction of the RV using the CARTOSOUND module and UNIVU system (Biosense Webster) was employed.

**Figure 2:**
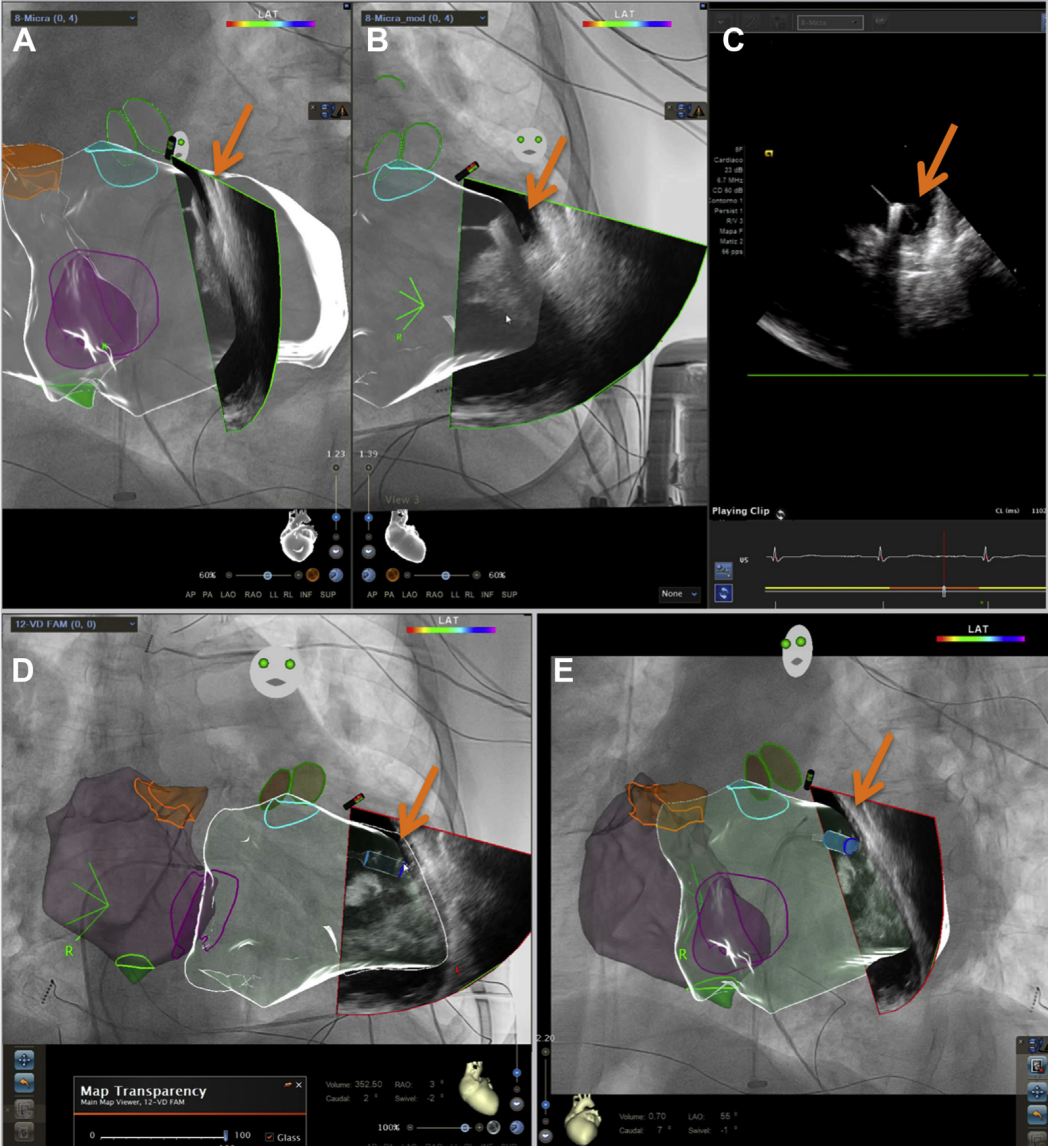
**A, B:** Intracardiac imaging catheter (ICE) combined with 3-dimensional ultrasound reconstruction of the right ventricle (RV) using the CARTOSOUND (Biosense Webster, Diamond Bar, CA) in right anterior oblique (RAO) and left anterior oblique (LAO) view. **C:** ICE view only. **D, E:** Catheter combined with 3-dimensional ultrasound reconstruction of the RV using the CARTOSOUND in RAO and LAO view, with graphic enhancement for clear identification of leadless pacemaker (LPM). (*Arrows* indicate position of LPM.)

Under ultrasound guidance, right femoral puncture was performed for the LPM introducer (27F) and a left femoral puncture for the ICE (9F). The introducer sheath was advanced to the level of the RA. A 3-D anatomical map including RA and RV chambers was subsequently created by means of the ICE 3-D ultrasound CARTOSOUND module and the UNIVU system. The Amplatz Gooseneck snare 10 mm loop diameter (Medtronic, Minneapolis, MN) was placed over the delivery system and through the sheath. Once it was in the right chambers, the traction on the snare system improved the reach of the delivery system by providing an additional point of contact beyond the superior aspect of the tricuspid valve and facilitated the reach of the delivery system in the enlarged chambers.^1^ The Amplatz Gooseneck snare was then removed after placing the delivery system in the RV (Figure 1D, Supplemental Video 1). After precise anatomical delineation performed with the ICE CARTOSOUND and the UNIVU system assistance, the LPM was deployed initially in the apex, where hypertrophic trabeculae were observed. At that location a high threshold was present and the device was not properly fixed, so it was relocated and deployed in a non–trabecular septal area (Supplemental Video 2). Adequate electrical parameters were then obtained, with an R-wave amplitude of 14.4 mV, a threshold of 0.63 V / 0.24 ms, and impedance of 880 ohms (Figure 2A and 2B). In the “pull-and-hold” test, seating of at least 3 tines was confirmed. The thread was cut and the LPM delivery system was withdrawn with no incidents.

**Figure 3:**
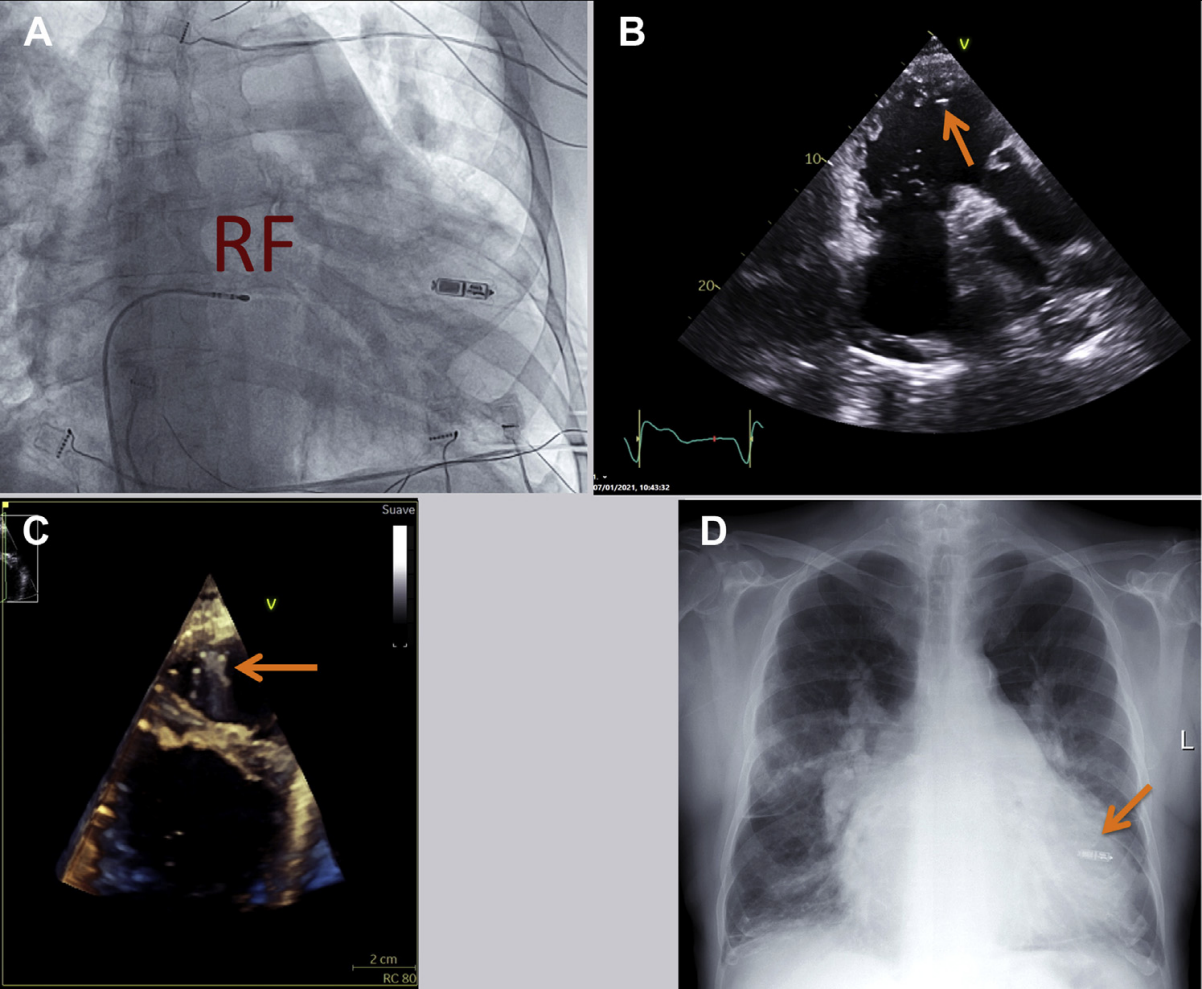
**A:** Fluoroscopic right anterior oblique view during atrioventricular node ablation after leadless pacemaker (LPM) implantation. *Arrow* indicates position of LPM. RF = ablation catheter. **B:** Echocardiography modified right 3-chamber view after LPM implantation. *Arrow* indicates position of LPM. **C:** Three-dimensional echocardiography, right ventricle view. *Arrow* indicates LPM position with identification of 4 tines. **D:** Posteroanterior thoracic radiograph after LPM implantation in midseptal location. *Arrow* indicates position of LPM.

Finally, as previously reported,^2^ through the 27F sheath, a 14F sheath was inserted and, through it, a ThermoCool (Biosense Webster) 4 mm ablation catheter was advanced to the right chambers. Radiofrequency ablation of the AV node was performed by standard techniques (Figure 3A) guided by the electroanatomical CARTO system. Control 2-D and 3-D echocardiogram and radiography were performed 24 hours after the procedure without complications (Figure 3B, 3C, and 3D, and Supplemental Video 3).

## Discussion

SVASD is a rare adult congenital heart disease that constitutes 5%–10% of all atrial septal defects. Atrial fibrillation or atrial flutter is an age-related reflection of atrial dilation and stretch, which causes substantial symptoms.^3^ In older patients, such as in this case, right heart failure is associated with tricuspid regurgitation and pulmonary arterial hypertension, conditions that make difficult both a pacemaker implantation and an AV node ablation.

Results of the Micra Investigational Device Exemption study and Micra Post-Approval Registry and mid-term to long-term results in a real-world setting have demonstrated excellent safety and efficacy performance,^2^ but its use is still challenging in patients with certain clinical conditions such as congenital heart diseases that might make LPM implantation difficult.^4^^,^^5^ Considering the alterations in the anatomy of the case, it is important to recognize the anatomical heart structures, the reason why an electroanatomical mapping system such as CARTO with UNIVU module was considered. This approach has demonstrated significantly reduced total fluoroscopy time and mean radiation dose, embracing safety and reproducibility.^6^ Additionally, CARTOSOUND image acquisition performed with real-time ICE has shown to improve image integration, enabling the depiction of the ultrasonic contours.^7^ Moreover, as described by Alyesh and colleagues^1^ in patients with very large right heart anatomy, we consider the use of a gooseneck snare, which was placed over the delivery system and through the sheath in order to improve the reach of the delivery system.^8^ We recommend that the snare should be retired as soon as the LPM delivery system is in the RV near the final position. From our point of view, this is a relevant asset because the lack of compliance of the silicone valve can lead to unnecessary bleeding as well as the possibility of entrapment with the strings in case of recapture of the LPM. Once the right heart anatomy reconstruction is performed and the device has been advanced to the RV, it is important to identify an adequate location for the deployment of the device by means of the CARTOSOUND system,^9^ especially in cases in which a marked hypertrabeculation can make LPM deployment difficult. AV node ablation could also be a difficult procedure in patients with right ventricle dilation, so this approach can also be of help at the time of AV node ablation.

## Conclusion

We present, to the best of our knowledge, the first case in which a hybrid approach combining image integration using intracardiac echography and 3-D reconstruction for leadless pacemaker implantation and a concomitant AV node ablation in a patient with an unrepaired congenital heart disease.

**Key Teaching Points**

• Unrepaired sinus venosus atrial septal defect is a rare adult congenital heart disease.
• Leadless pacemaker (LPM) and atrioventricular node ablation can be performed in the same procedure, including in complex cases, with no additional risk.
• Considering the complexity of the case, an implantation of an LPM poses multiple challenges, so a hybrid procedure with multimodality imaging including a tridimensional intracardiac imaging catheter reconstruction with the CARTOSOUND module and UNIVU image integration system (Biosense Webster, Diamond Bar, CA) can be useful.
• “Optimized viewing” of LPM deployment in the best zone assures the electrical performance, avoiding trabeculated right ventricle (poor stability at higher thresholds) and instability of implant.
• A future exhaustive, comprehensive design should include a complete echocardiography evaluation of the conditions that could make the LPS implantation difficult, such as chamber diameters, tricuspid severity, or hypertrophic trabeculae.

## Appendix Supplementary data

Video 1 Fluoroscopic LPM implantation.

Video 2 CARTOSOUND UNIVU-ICE during LPM implantation.

Video 3 3-D echocardiography right ventricle view.
