# Supplementary material for: Leadless pacemaker implantation with hybrid image mapping technique in a congenital heart disease case
Source: HeartRhythm Case Rep. 2021 Jul 18;7(12):797–800. doi: 10.1016/j.hrcr.2021.07.004 (PMC8695296; doi:10.1016/j.hrcr.2021.07.004)
Supplement: Supplementary material file [file mmc4.docx]

**VIDEOS**

Video 1: Fluoroscopic LPM implantation.

Video 2: CARTOSOUND UNIVU-ICE during LPM implantation.

Video 3: 3-D Echocardiography right ventricle view.
